# Psychological care of caregivers, nurses and physicians: a study of a new approach

**DOI:** 10.1002/cam4.163

**Published:** 2014-01-04

**Authors:** Maurizio S Abeni, Margherita Magni, Martina Conte, Silvia Mangiacavalli, Lara Pochintesta, Gaia Vicenzi, Virginia V Ferretti, Alessandra Pompa, Federica Cocito, Catherine Klersy, Alessandro Corso

**Affiliations:** 1 Division of Hematology, Fondazione IRCCS Policlinico San Matteo, University of PaviaPavia, Italy; 2 Service of Biometry and Statistics, Fondazione IRCCS Policlinico San Matteo, University of PaviaPavia, Italy

**Keywords:** Cancer patients, caregiver, myeloma, nurse, physicians, psychological care

## Abstract

There is much evidence demonstrating that psychosocial interventions in caregivers and oncological staff produce an improvement in their patients' quality of life. The aim of this explorative study was to evaluate the effect of a new approach in promoting more functional ways to face stressful situations in the constellation of people around patients: caregivers, physicians and nurses. Thirty-four subjects were divided into three groups: 10 caregivers, 11 physicians, and 13 nurses. A “Balint Group” method modified according to a mindfulness technique was used as the intervention. Three assessment tools were administered to the participants at baseline, during, and after completion of the study: the Response Evaluation Measure (REM-71), the Satisfaction Profile (SAT-P), and the Group Climate Questionnaire (GCQ). Mean values of defense mechanisms determined by the REM-71 were compared with those of the standard population. At baseline, we observed a prevalence of immature defenses in the three groups, with mean values above those in the standard population. After the psychological intervention, a tendency to normalization of the mean values was observed, indicating the development of more adaptive ways of using defense mechanisms and the effectiveness of the intervention. Group climate, assessed through the GCQ, showed an increase in the “Engagement” factor and a decline in the “Conflict” factor in all groups. This study suggests that group treatment focused on changing personal responses to stressful situations can induce more adaptive strategies enabling caregivers, hematologists, and nurses to help patients better and thereby improve their quality of life.

## Introduction

There is growing consensus that psychosocial support should be integrated into the routine care of patients with cancer 1–4. A more recent concept is to extend this support to the patients' physicians, nurses, and caregivers (husbands, wives, or relatives) 5,6.

Patients tend to focus on their illness and their body. However, their emotional burden often causes the adoption of strong dysfunctional defense mechanisms that can bring them to deny the idea of being ill and consequently to refuse or not seek the help of medical staff 7,8. Denial and negation defenses can delay the diagnosis and reduce adherence to treatment and follow-up 9–12. Caregivers can be the link between patients and physicians, supporting and creating a good alliance between the two that is considered essential for patients to overcome dysfunctional defense mechanisms. Psychosocial intervention for caregivers can be of paramount importance in improving the quality of life of patients and their families 13,14, especially in terms of better global functioning.

Patients and caregivers are not, however, the only protagonists of the battle against cancer. Oncologists and nurses deal with existential questions of life and death daily, which are always difficult issues to face and manage. It has been demonstrated that medical oncologists are more prone to experience symptoms of depression than other internal medicine physicians and this phenomenon can be observed even during their training 15–17. If these difficulties are not recognized promptly they can lead to burnout, a syndrome characterized by a loss of enthusiasm for work, a feeling of cynicism, and a low sense of personal accomplishment, which can cause medical errors and malpractice 18,19. Other factors can contribute to the development of a burnout: excessive workload, a sense of impotence, frustration, limited autonomy, and a perception that one's own work is meaningless. These factors might further reduce autonomy and efficiency, increase workload, and reduce the willingness to interact with patients, thereby worsening the relationship between patients and medical staff 20,21. This is an important point as one of the causes of depression in patients is a poor relationship with physicians, or in general with all the oncological staff 22. Emotional distress is also a strong predictor of poor self-management and high health care costs.

Given the prevalence of distress among oncologists and its implications for patients and their care, it could be important to make physicians undergo periodic screening or monitoring for burnout particularly because they often do not look for help. There is now considerable evidence that any intervention in oncological staff and caregivers increases their capacity to bear emotional burdens and consequently improves the patients' quality of life 23.

Starting from these observations, we decided to focus on the “constellation” of people (nurses, physicians, and caregivers) around patients affected by multiple myeloma and designed a pilot study. The project, conducted in the three groups, was focused on the identification of personal responses to stressful situations (defense mechanisms) evaluated through several assessment tools administered to the participants at baseline and after completion of the psychological intervention. The identification of defense mechanisms against illness is of the utmost importance: on the one hand, these mechanisms represent a way of coping with anxiety triggered by threat, on the other hand, they allow people to establish new ways of relating with the world and with themselves. The aim of this explorative study was to evaluate the effect of a group intervention in improving caregivers' knowledge and quality of life, and in promoting among physicians and nurses a different way of facing the difficulties of their job.

## Methods

### Participants and interventions

An explorative study was carried out between caregivers, physicians and nurses of Policlinico San Matteo of Pavia, Italy. Hematologists and nurses participated voluntarily; the caregivers invited to participate were sampled stratifying for phases of disease (onset, relapse, advanced phase). The number of participants from each group who dropped out during the study was recorded (early and late dropouts). The study was approved by the Ethics Committee of Policlinico San Matteo of Pavia, and all participants signed a written informed consent.

The research was divided into three phases: the preintervention phase (T0), the intervention period, and the follow-up (T1).

In the preintervention phase, individual interviews, of 50 min each, were conducted by a clinical psychologist to introduce the topic and the objective of the research, to administer the questionnaires response evaluation measure (REM-71), Satisfaction profile (SAT-P), and to investigate the present level of personal satisfaction and stress. The questionnaires, anonymous and self-compiled, were the same for all participants.

In the second phase of the study, the intervention period, each group (caregivers, physicians, nurses) was planned to have 30 sessions, each of 60 min, using a “Balint Group” method 24,25, modified according to a mindfulness technique 26,27. This method promotes a group process of exploration and training based on reflection about relational experiences with patients, focusing on development of attentive awareness of sensations, thoughts, and perceptions of reality at the time of the session. The group leader presented the topic of each discussion. At the end of each session, an anonymous questionnaire group climate questionnaire (GCQ) was administered to all participants.

In the final phase (T1), at the end of all sessions (after 1 year), the same procedure as in the preintervention phase was used.

### Assessment tools

The research was focused on the identification of personal responses to stressful situations and then on the development of more adaptive ways of managing these situations. The assessment tools (REM-71, SAT-P, and GCQ) were not specific for the populations in the study.

#### Response evaluation measures-71

The REM-71 28 is a self-report questionnaire to assess defense mechanisms in adults through the use of 71 items, measured on a Likert scale from 1 (complete disagreement) to 9 (complete agreement). The scale defines defenses as self-regulatory processes that could be considered metrics of positive psychological health. The theoretical approach of the instrument implies a developmental model of defenses placed along a continuum of maturity–immaturity. The scale's validity is supported by several studies involving adults, adolescents, and school-aged children. Defenses were grouped into two factors: Factor 1 (*immature defenses*) and Factor 2 (*mature defenses*) 29 (Table 1). Factor 1 comprises 14 defenses that distort reality in accordance with expected outcomes, leading to less adaptive functioning. Factor 2, by contrast, comprises seven defenses that attenuate unwelcome reality, allowing more adaptive functioning. We considered estimated values ≥4.4 30 as indicating a potentially dysfunctional way of using defense mechanisms. Mean values of defense mechanisms obtained through questionnaires were compared with those of the standard population 30.

**Table 1:** Description of defense mechanisms.

	Description	Sample item
*Factor 1*		
“Immature defenses”	The individual deals with emotional conflicts or internal or external stressors…	
Acting out	… by actions rather than reflections or feelings	When I am upset I do things without thinking
Splitting	… by viewing himself or herself or others as all good or all bad, failing to integrate the positive and negative qualities of the self and others into a cohesive images	When someone I like lets me down, I usually trust them again
Displacement	… by generalizing or redirectioning a feeling about one object onto another, usually less-threatening object	I won't let people in authority know I'm angry at them, but everyone else better watch out!
Dissociation	… by a temporary alteration in the integrative functions of consciousness, memory, perception of self or the environment, or sensory/motor behavior.	I often get the feeling that whatever is going on is not really happening to me
Fantasy (Autistic)	… by excessive daydreaming as a substitute for human relationship, more direct and effective action, or problem solving	I like to imagine that my life is very different.
Passive aggression	… by indirectly, unassertively, and often self-detrimentally expressing aggression toward others. There is a facade of overt compliance masking covert resistance, resentment, or hostility	If someone is unfair to me I probably won't do what I told them I'd do.
Projection	… by falsely attributing to another his or her own unacceptable feelings, impulses, or though	I am usually treated unfairly.
Repression	… by being unable to remember or unable to be cognitively aware of disturbing wishes, thoughts, or experiences	When I should have strong feelings, I don't' feel anything
Omnipotence	… by feeling or acting as if he or she possesses special powers or abilities and is superior to others	I don't want to brag, but usually I'm the one who knows how to get things done
Undoing	… by words or behavior designed to negate or to make amends symbolically for unacceptable thoughts, feelings, or actions	I repeat special thoughts or words over and over to myself when I am uptight or frightened
Conversion	… by convert mental conflict to a physical symptom	Sometimes I have lost all the feeling in one part of my body and nobody could explain why
Somatization	… by the expression of psychological conflict via bodily symptoms without symbolic content	When I get stressed I get ill really easily
Withdrawal	… by the retreat from reality, and removal of self from usual social discourse	When things upset me I'd rather be by myself
Suppression	… by intentionally avoid thinking about disturbing problems, desires, feelings or experiences	When I need to, I can put my problems on hold until later when I can think about them.
*Factor 2*		
“Mature defenses”	The individual deals with emotional conflicts or internal or external stressors…	
Denial	… by refusing to acknowledge some painful aspect of external reality or subjective experience that would be apparent to others	When I am upset I remind myself that everything is really okay
Humor	… by emphasizing the amusing or ironic aspects of the conflict or stressor	When things go wrong, I can still see the funny side
Intellectualization	… by the excessive use of abstract thinking or the making of generalizations to control or minimize disturbing feelings	I use reason and logic, not feelings, to understand people
Reaction formation	… by substituting behavior, thoughts, or feelings that are diametrically opposed to his or her own unacceptable thoughts or feelings	Often I act really nice when actually I am pretty upset
Idealization	… by attributing exaggerated positive qualities to self or other	I know this great person whose advice I can usually trust
Altruism	… by caring of others needs in order to satisfy his own	I go out of my way to help people
Sublimation	… by channeling potentially maladaptive feelings or impulses into socially acceptable behavior	I like to write stories or poems when I've just been through a really rough situation

#### Satisfaction profile

The SAT-P 31 is a self-report scale widely used in the health care context. The scale defines satisfaction as the result of a cognitive process that compares real with ideal expectations. Level of satisfaction is linked to the gap between real and ideal expectations. The SAT-P assesses subjective satisfaction in the preceding month using five macro-categories: psychological and physical functioning, work, sleep/diet/free time, and social functioning. The questionnaire consists of 32 items, measured on a visual analog scale: the level of agreement with a statement is indicated by marking a position along a continuous line (10 cm) between two end-points (totally satisfied/totally dissatisfied). It assesses a satisfaction profile that should be integrated with clinical data.

#### Group climate questionnaire—short version

The GCQ 32,33 is a self-report questionnaire consisting of 12 items rated on a 6-point scale, from 1 = not at all to 6 = extremely. It measures how members consider their group experience through three scales: engagement, conflict, avoidance. Engagement is the degree of cohesion and work orientation of the group. Avoidance is the degree to which individuals rely on group members and leaders. Conflict consists of the subscales friction, distrust, and mutual withdrawal. The GCQ was administered after every group session.

### Statistical analysis

Data were described as means and standard deviations (SD), if continuous, and as counts and percentages, if categorical. Within-subject comparisons of scores were performed by means of a general linear regression model for repeated measures, with calculation of robust standard errors to account for within-subject correlation. Mean changes and 95% confidence intervals (95% CI) were computed. The association between GCQ scores and number of sessions was assessed with Spearman's rho coefficient. For this analysis, data were anonymous and were pooled across groups.

Stata 12 34 was used for statistical computations. A two-sided *P*-value <0.05 was considered statistically significant.

## Results

Thirty-four subjects were enrolled in the study (10 caregivers, 11 physicians, and 13 nurses). Four participants (two caregivers, one physician, and one nurse) dropped out the study, three within the first 3 months (early dropout) and one after 3 months (late dropout). The three early dropouts, one caregiver, one physician, and one nurse, stopped participating for employment reasons; the other caregiver (the late dropout) withdrew from the study because of the worsening clinical condition and unbearable increased care of the relative.

### Caregivers

**Figure 1:**
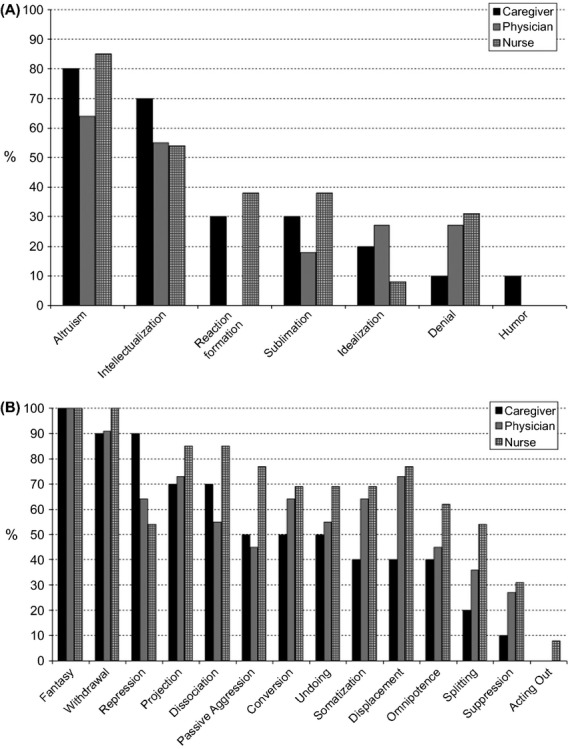
Estimated percentage of mature (A) and immature defenses (B) in the three groups, at baseline.

At baseline, the frequency of immature defenses was higher than that of mature ones. As shown in Figure 1B, the most frequent (≥50%) immature mechanisms were: fantasy (100%), withdrawal (90%), repression (90%), projection (70%), dissociation (70%), passive aggression (50%), conversion (50%), and undoing (50%). The only two mature mechanisms were altruism (80%) and intellectualization (70%) (Fig. 1A).

The comparison of the mean values of defense mechanisms in caregivers at baseline with those of the standard population (Table 2) showed higher values for four immature defenses: fantasy, conversion, projection, and repression.

**Table 2:** Mean scores of the standard population and participants of all three groups at baseline, prior to the intervention.

Defenses	Adult population (*n* = 543)	Caregivers T0 (*n* = 10)	Hematologists T0 (*n* = 11)	Nurses T0 (*n* = 13)
Immature defenses				
Acting out	3.63 ± 1.63	1.00 ± 0.00	1.97 ± 1.57	1.97 ± 1.57
Splitting	5.24 ± 1.69	3.85 ± 2.10	4.77 ± 1.88	4.77 ± 1.88
Displacement	3.06 ± 1.61	5.85 ± 1.75	5.72 ± 1.61	5.72 ± 1.61
Dissociation	3.14 ± 1.68	4.27 ± 2.72	5.67 ± 2.08	5.67 ± 2.08
Fantasy	3.44 ± 1.84	7.73 ± 0.76	8.00 ± 0.68	8.00 ± 0.68
Passive aggression	3.99 ± 1.45	4.18 ± 2.21	5.31 ± 1.55	5.31 ± 1.55
Projection	2.20 ± 1.22	5.68 ± 2.09	5.75 ± 1.15	5.75 ± 1.15
Repression	3.52 ± 1.75	6.18 ± 2.40	4.77 ± 1.87	4.77 ± 1.87
Omnipotence	4.45 ± 1.44	4.58 ± 1.99	5.33 ± 1.58	5.33 ± 1.58
Undoing	3.60 ± 1.81	4.98 ± 1.62	5.10 ± 1.03	5.10 ± 1.03
Conversion	1.26 ± 0.78	5.33 ± 2.14	5.51 ± 1.38	5.51 ± 1.38
Somatization	4.16 ± 1.98	5.18 ± 3.06	4.92 ± 1.67	4.92 ± 1.67
Withdrawal	5.58 ± 2.05	5.85 ± 1.21	6.58 ± 0.91	6.58 ± 0.91
Suppression	3.98 ± 2.13	3.15 ± 1.82	3.79 ± 1.27	3.79 ± 1.27
Mature defenses				
Denial	4.19 ± 1.70	3.38 ± 1.07	3.86 ± 0.96	3.86 ± 0.96
Humor	5.15 ± 1.69	2.64 ± 1.21	2.36 ± 0.83	2.36 ± 0.83
Intellectualization	4.63 ± 1.37	5.03 ± 2.71	5.25 ± 1.93	5.25 ± 1.93
Reaction formation	4.00 ± 1.56	2.85 ± 0.75	3.77 ± 1.46	3.77 ± 1.46
Idealization	6.00 ± 1.82	3.15 ± 1.43	3.15 ± 1.36	3.15 ± 1.36
Altruism	7.35 ± 1.18	5.00 ± 2.76	6.38 ± 1.66	6.90 ± 2.73
Sublimation	5.19 ± 1.62	3.64 ± 1.15	4.75 ± 1.25	3.90 ± 2.10

**Figure 2:**
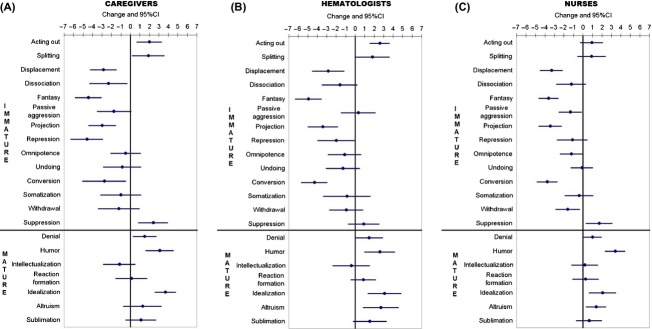
Changes between, before, and after treatment and 95% confidence intervals, in the three groups: caregivers (A), hematologists (B), and nurses (C).

The follow-up analysis performed after the intervention showed a general tendency to normalization with a significant increase in mature defense mechanisms (such as humor, idealization, and denial) and a decrease in many immature defense mechanisms, in particular, those that had been found to be significantly higher with respect to the standard population (fantasy, repression, projection, and conversion). We also observed an increase in one immature defense: suppression. Changes between before and after the group intervention and the 95% CI are illustrated in Figure 2A.

At baseline, SAT-P results were all within the range of the standard population. This finding conflicted with the information that emerged from the individual interviews which revealed remarkable difficulties in managing everyday life. After the intervention, all scores for quality of life perception increased by 10%–20%, although this improvement just fell short of being statistically significant (Table 3).

**Table 3:** Satisfaction profile scores of the three groups before (T0) and after (T1) treatment, with associated 95% confidence intervals and statistical significance.

	Caregivers				Hematologists				Nurses			
Factors	T0	T1	Change (95% CI)	*P*	T0	T1	Change (95% CI)	*P*	T0	T1	Change (95% CI)	*P*
Psychological function	66.99 ± 10.31	76.63 ± 10.30	9.64 (−0.97 to 20.24)	0.07	66.68 ± 13.36	62.59 ± 11.80	−4.09 (−16.62 to −8.44)	0.50	70.60 ± 14.94	64.06 ± 15.93	−6.54(−11.55 to 10.93)	0.33
Physical function	53.07 ± 15.13	64.84 ± 17.28	11.77 (−6.31 to 29.85)	0.18	57.93 ± 10.55	51.59 ± 10.82	−6.34 (−16.64 to 3.97)	0.21	57.36 ± 14.12	51.28 ± 17.80	−6.08 (−20.35 to 8.19)	0.38
Job	63.02 ± 23.67	68.69 ± 23.79	5.66 (−19.78 to 31.10)	0.64	66.38 ± 21.87	63.18 ± 17.21	−3.20 (−21.04 to 14.64)	0.71	66.22 ± 18.17	61.67 ± 10.91	−0.54 (−12.62 to 11.54)	0.93
Sleep/eating/spare time	62.24 ± 15.03	73.31 ± 10.51	11.07 (−2.06 to 24.21)	0.09	47.78 ± 11.87	50.24 ± 15.70	2.46 (−10.29 to 15.21)	0.69	55.31 ± 15.95	49.67 ± 17.84	−5.64 (−19.70 to 8.42)	0.41
Social function	71.93 ± 22.76	79.62 ± 12.29	7.69 (−11.33 to 26.71)	0.40	76.39 ± 13.82	76.23 ± 14.01	−0.16 (−12.85 to 12.52)	0.98	63.46 ± 20.46	62.45 ± 16.27	−1.02 (−16.28 to 14.25)	0.89

As regards the GCQ (assessed at each session), the number of followed sessions showed a direct correlation with the “Engagement” factor and an inverse correlation with the “Conflict” factor (Table 4).

**Table 4:** Climate group significance level and Spearman's correlation coefficient. *n* values represent the total number of intervention sessions for each group.

	Caregivers (*n* = 24)		Hematologists (*n* = 33)		Nurses (*n* = 33)	
Variable	Rho	*P*	Rho	*P*	Rho	*P*
Engagement	**0.2340**	**0.0322**	0.0548	0.5361	0.0533	0.5244
Conflict	**−0.3060**	**0.0044**	−0.1006	0.2546	−0.0709	0.3921
Avoidance	0.0937	0.3997	−0.1619	0.0658	−0.1139	0.1867

Values in bold are statistically significant.

### Hematologists

The baseline, preintervention data revealed a predominance of immature strategies (Fig. 1B): fantasy (100%), withdrawal (90%), projection (73%), displacement (73%), somatization (64%), conversion (64%), repression (64%), dissociation (54%), and undoing (54%). The only two mature defenses were altruism (64%) and intellectualization (54%) (Fig. 1A).

The comparison with the standard population (Table 2) showed that hematologists, like the caregivers, prevalently used immature defense mechanisms, in particular: fantasy, conversion, and projection.

The analysis after the intervention highlighted a significant increase in mature defenses (such as humor, altruism, idealization, and denial), and a decline of many immature defenses, in particular, those identified as being significantly more frequent with respect to the standard population: fantasy, conversion, and projection. There was clear evidence of a general tendency to normalization for all defenses. Changes between before and after the group intervention and the 95% CI are illustrated in Figure 2B.

The preintervention evaluation of perceived quality of life showed the same discrepancy as in the group of caregivers between what was picked up by the SAT-P results, which were all in the standard population average, and what emerged from the interviews. In this group, the change from baseline was very close to zero, and far from being statistically significant (Table 3).

Climate group evaluation showed a direct correlation between the number of followed sessions and the “Engagement” factor, and an inverse correlation between the number of followed sessions and “Conflict” and “Avoidance” factors (Table 4). However, these relations were not statistically significant.

### Nurses

A high frequency of immature defenses was observed also in this group (Fig. 1B): fantasy (100%), withdrawal (100%), dissociation (85%), projection (85%), passive aggression (77%), displacement (77%), somatization (69%), conversion (69%), undoing (69%), omnipotence (61%), repression (54%), and splitting (54%). Only two mature defenses were present: altruism (85%) and intellectualization (54%) (Fig. 1A). The mean values for some of the defenses (fantasy, projection, and conversion) were higher than those in the standard population (Table 2).

In line with the results of the other groups (caregivers and hematologists), at follow-up, mature defenses (humor, altruism, idealization, and denial) increased significantly, while immature defenses tended to decrease. Only one immature defense increased: suppression. Changes between before and after the intervention and their 95% CI are reported in Figure 2C.

Before the intervention, the SAT-P results were all similar to those in the standard population. As for the hematologists, there were virtually no changes in the SAT-P score in nurses after the intervention (Table 3). Once again, there was an evident discrepancy between the results of the questionnaire and the interviews.

As for the hematologists, the number of followed sessions showed a direct correlation with the “Engagement” factor and inverse correlations with “Conflict” and “Avoidance” factors, but these relationships were not statistically significant (Table 4).

## Discussion

The emotional burden of caregivers has usually been characterized in previous studies as creating anxiety and depression with a low quality of life (both physical and psychological). For nurses and physicians who deal with oncological diseases, most studies have focused on burnout and its personal (anxiety, depression) and professional (malpractice) consequences. In both cases, well-being and personal growth were measured in terms of specific symptoms and consequently any therapeutic approaches were addressed at eliminating these symptoms.

In contrast, the new approach adopted in this explorative study arose from the belief that a reintegration of personal satisfaction cannot be obtained only from reducing symptoms or improving quality of life, but requires the development of more functional and mature ways of facing reality and its stressful situations, such as severe illness.

The analysis performed at baseline, prior to the intervention, showed a high frequency of immature defenses in all three groups: this implies less functional and adaptive ways of facing everyday situations and a low acceptance of reality. This was confirmed by the comparison of our sample with the standard population; all three groups showed, as a common finding, three immature defenses: fantasy, conversion, and projection. The use of these mechanisms of defense is consistent with evoking a death fantasy as a consequence of a confrontation with a severe illness. Conversion is a defensive response to death and contamination anxiety evoked by illness. It could be considered as an unconscious identification with the sick role, reproducing somatic symptoms observed in patients to reduce stress. Projection is a way of transferring unacceptable feelings, such as anger, to other people, that is turning the inner danger into an outer one. For example, the hospital environment could be perceived as constantly hostile and dangerous. Fantasy is a way of coping with problems and situations that evoke great impotence through an excessive use of daydreaming as a substitute for human relationships, more direct and effective action, or problem solving. The frequent use of fantasy as a defense in this context could also embellish the interpretation of somatic symptoms, leading to the idea of being affected by a serious illness. Contrariwise, the use of repression as a defense mechanism, which was more common in caregivers than in the standard population, could lead to an inability to remember diseases, shifting attention to specific symptoms of illness or to drug-related side effects.

The effect of the psychological intervention was evident in all groups. We observed a decrease of immature defenses, including those that were more present in our study groups at baseline than in the standard population and an increase in mature defenses *(*humor, idealization, and denial) after the intervention. Altruism increased among physicians and nurses especially in solidarity and cooperation with the colleagues. By contrast, altruism remained stable in caregivers, due to its high value at baseline. An increase in mature defenses implies a greater ability to accept and mitigate the unpleasant aspects of reality, with consequent better self-management.

This trend to normalization in the use of defense mechanisms, in common to all the groups, is a clear indication of the effect of the intervention. Of note, in caregivers and nurses, suppression, which was within the normal range for standard adults at baseline, increased after the intervention. This could be due to the short duration of the intervention and so it might indicate a transitional stage, as already well described in psychotherapy literature. This phenomenon is explained by the fact that defense mechanisms are structures that change slowly, requiring time to develop and become stabilized 35.

During the intervention, cohesion increased in all three groups and the avoidance of personal responsibility of work group decreased. Cohesion is comparable to therapeutic alliance in individual psychotherapy 36. Our approach was also effective in creating a good group climate focused on more productive cooperation and support. However, although significant, the correlation was weak.

Quality of life perception did not change significantly after the invention, except for a relevant, but not statistically significant, increase in scores in the caregiver's cohort only. This could be due to the caregivers' greater attendance at sessions, which implied reinforcement of the group treatment, in line with the GCQ results.

In conclusion, this study suggests that a work group, promoting sharing and collaboration, leads to the induction of mature defenses enabling reality to be faced in a more adaptive and effective way. As defense mechanisms are unconscious functions, once established, people keep on using them in their everyday life. The development of more adaptive strategies can help caregivers to accept their new stressful condition, and consequently provide better assistance for the people they are caring for, and can help hematologists and nurses foster better ways to face frustration and impotence, in order to prevent burnout and facilitate their relationships with patients. Therefore, results of this study offer a useful starting point for the planning and the development of future studies with the aim to evaluate the efficacy of the “Balint Group” modified method on caregivers' quality of life and on the ability of physicians and nurses to deal with stressful situations.

## Conflict of Interest

None declared.

## References

[b1] Fann JR, Ell K, Sharpe M (2012). Integrating psychosocial care into cancer services. J. Clin. Oncol.

[b2] Jacobsen PB, Wagner LI (2012). A new quality standard: the integration of psychosocial care into routine cancer care. J. Clin. Oncol.

[b3] Kissane DW, Bylund CL, Banerjee SC, Bialer PA, Levin TT, Maloney EK (2012). Communication skills training for oncology professionals. J. Clin. Oncol.

[b4] Northouse L, Williams A, Given B, McCorkle R (2012). Psychosocial care for family caregivers of patients with cancer. J. Clin. Oncol.

[b5] Nijboer C, Triemstra M, Tempelaar R, Sanderman GAM, Van den Bos (1999). Determinants of caregiving experiences and mental health of partners of cancer patients. Cancer.

[b6] Glajchen M (2004). The emerging role and needs of family caregivers in cancer care. J. Support Oncol.

[b7] McGuire GP, Stoll BA (1979). The will to live in cancer patients. Mind and cancer prognosis.

[b8] Stoll BA, Stoll BA (1979). Defining the subject of inquiry. Mind and cancer prognosis.

[b9] Goldberg RJ (1983). Systematic understanding of cancer patients who refuse treatment. Psychother. Psychosom.

[b10] Green JA (1983). Compliance and cancer chemotherapy. Br. Med. J.

[b11] Lewis C, Linet MS, Abelof MD (1983). Compliance with cancer therapy by patients and physicians. Am. J. Med.

[b12] Tamaroff MH, Festa RS, Adesman AR, Walco GA (1992). Therapeutic adherence to oral medication regimens by adolescents with cancer. Clinical and psychologic correlates. J. Pediatr.

[b13] Hudson P (2004). A critical review of supportive interventions for family caregivers of patients with palliative-stage cancer. J. Psychosoc. Oncol.

[b14] Martire LM, Lustig AP, Schulz R, Miller GE, Helgeson VS (2004). Is it beneficial to involve a family member? A meta-analysis of psychosocial interventions for chronic illness. Health Psychol.

[b15] Bellini LM, Baime M, Shea JA (2002). Variation of mood and empathy during internship. Jama.

[b16] Shanafellt TD (2005). Finding meaning, balance and personal satisfaction in the practice of oncology. J. Support Oncol.

[b17] Brazeau CM, Schroeder R, Rovi S, Boyd L (2010). Relationship between medical student burnout, empathy, and professionalism climate. Acad. Med.

[b18] Shanafelt TD, Bradley KA, Wipf JE, Back AL (2002). Burnout and self reported patient care in an internal medicine residency program. Ann. Intern. Med.

[b19] West CP, Huschka MM, Novotny PJ, Sloan JA, Kolars JC, Habermann TM (2006). Association of perceived medical errors with resident distress and empathy: a prospective longitudinal study. Jama.

[b20] DiMatteo MR, Sherbourne CD, Hays RD, Ordway L, Kravitz R, McGlynn EA (1993). Physicians' characteristics influence patients' adherence to medical treatment: results from the medical outcomes study. Health Psychol.

[b21] Haas JS, Cook EF, Puopolo AL, Burstin HR, Cleary PD, Brennan TA (2000). Is the professional satisfaction of general internists associated with patient satisfaction?. J. Gen. Intern. Med.

[b22] Shanafelt TD, Dyrbye L (2012). Oncologist burnout: causes, consequences and responses. J. Clin. Oncol.

[b23] Linn LS, Brook RH, Clark VA, Davies AR, Fink A, Kosecoff J (1985). Physician and patient satisfaction as factors related to the organization of internal medicine group practices. Med. Care.

[b24] Balint M (1961). Medico, paziente e malattia.

[b25] Luban-Plozza B (1986). I gruppi balint: un metodo formativo alla relazione. formazione psicologica del medico, degli operatori sanitari e scolastici.

[b26] Siegel DJ (2009). Mindfulness e cervello.

[b27] Mace C (2010). Mindfulness e salute mentale.

[b28] Steiner H, Araujo KB, Koopman C (2001). The response evaluation measure (REM-71): a new instrument for the measurement of defenses in adult and adolescent. Am. J. Psychiatry.

[b29] Prunas A, Preti E (2007). Il defense style questionnaire: una review della letteratura. Proprietà psicometriche e struttura fattoriale. Psichiatria e Psicoterapia.

[b30] Prunas A, Madeddu F, Pozzoli S, Gatti C, Shaw R, Steiner H (2009). The Italian version of the response evaluation measure (REM-71). Compr. Psychiatry.

[b31] Majani G, Callegari S (1998). Satisfation profile (SAT-P).

[b32] MacKenzie KR, Dies RR, MacKenzie KR (1983). The clinical application of a group climate measure. Advances in group psychotherapy: integrating research and practice.

[b33] Costantini A, Picardi A (2002). Questionario sul clima di gruppo: validazione di una misura di processo per le psicoterapie di gruppo. Rivista di Psichiatria.

[b34] StataCorp (2011).

[b35] Gill MM (1985). Teoria e tecnica dell'analisi del transfert.

[b36] Kivlighan DM, Angelone EO (1992). Interpersonal problems: variables influencing participants' perception of group climate. J. Counseling Psychol.

